# TMEM205 induces TAM/M2 polarization to promote cisplatin resistance in gastric cancer

**DOI:** 10.1007/s10120-024-01517-2

**Published:** 2024-06-08

**Authors:** Qiang Fu, Xuwei Wu, Zhongqi Lu, Ying Chang, Quanxin Jin, Tiefeng Jin, Meihua Zhang

**Affiliations:** 1 Department of Health Examination Centre, Yanbian University Hospital, Yanji, 133002 China; 2 Department of Ultrasound Medicine, Affiliated Hospital of Yanbian University, Yanji, 133000 Jilin China; 3 Department of Pathology and Cancer Research Center, Yanbian University Medical College, Yanji, 133002 China; 4 Key Laboratory of the Science and Technology Department of Jilin Province, Yanji, China; 5 Department of Pathology, Chifeng Municipal Hospital, Chifeng, 024000 China; 6 Department of Immunology and Pathogenic Biology, Yanbian University Medical College, Yanji, China

**Keywords:** Gastric cancer, Transmembrane protein 205, Cisplatin resistance, Tumor-associated macrophages, Wnt/β-catenin

## Abstract

Cisplatin (DDP) is a basic chemotherapy drug for gastric cancer (GC). With the increase of DDP drug concentration in clinical treatment, cancer cells gradually became resistant. Therefore, it is necessary to find effective therapeutic targets to enhance the sensitivity of GC to DDP. Studies have shown that Transmembrane protein 205 (TMEM205) is overexpressed in DDP-resistant human epidermoid carcinoma cells and correlates with drug resistance, and database analyses show that TMEM 205 is also overexpressed in GC, but its role in cisplatin-resistant gastric cancer remains unclear. In this study, we chose a variety of experiments in vivo and vitro, aiming to investigate the role of TMEM 205 in cisplatin resistance in gastric cancer. The results showed that TMEM 205 promoted proliferation, stemness, epithelial–mesenchymal transition (EMT), migration and angiogenesis of gastric cancer cells through activation of the Wnt/β-catenin signaling pathway. In addition, TMEM205 promotes GC progression by inducing M2 polarization of tumor-associated macrophages (TAMs). These results suggest that TMEM205 may be an effective target to regulate the sensitivity of GC to DDP, providing a new therapeutic direction for clinical treatment.

**Supplementary Information:**

The online version contains supplementary material available at 10.1007/s10120-024-01517-2.

## Introduction

GC is the fifth and fourth highest malignant tumor in the world in terms of morbidity and mortality [1]. For the treatment of GC, DDP is one of the chemotherapy drugs for patients with postoperative and advanced, but its drug resistance affects the treatment effect [2]. Therefore, it is necessary to find an effective therapeutic target to enhance the sensitivity of GC cells to DDP.

As a member of the transmembrane proteins (TMEMs), TMEM205 is a newly discovered protein whose molecular and functional characterization is just beginning to be studied, and it constructed transmembrane channels for a variety of substances, mediating communication between the intracellular and extracellular environment [3] (molecular structure of TMEM205 is shown in Supplementary Fig. 1). TMEM205 overexpressed in DDP-resistant human epidermoid cancer cells and related to chemical resistance. Uksha Saini et al. demonstrated the mechanotransduction pathway of TMEM205-mediated chemoresistance and used oncolytic herpes simplex virus (oHSV) and cisplatin as an effective strategy for the treatment of ovarian clear cell carcinoma (OCCC). Oncolytic virus (oHSV) in combination with cisplatin inhibited the growth of OCCC tumors in immunodeficient and immunocompetent mouse models [4]. In addition, TMEM205 underwent nuclear translocation in DDP-resistant cells, and interacted with RAB8 to promote the accumulation of DDP outside the cell, enhancing DDP resistance [5, 6]. Related studies have shown that TMEMs were involved in cancer progression by promoted cancer cells proliferation, stemness, migration, angiogenesis, and EMT process. TMEM107 affected proliferation, migration, and EMT process of NSCLC cells [7]. Knocking down TMEM215 inhibited the angiogenesis of HUVECs [8]. TMEM119 enhanced stemness of breast cancer cells by activating the Wnt/β-catenin signaling pathway [9]. However, the effects of TMEM205 on GC stemness, migration, angiogenesis and EMT process are unclear.

Tumor-associated macrophages are the most abundant immune cells in tumor microenvironment (TME). TAMs can be polarized into classically activated M1 type (TAM/M1) and alternative activated M2 type (TAM/M2) under the stimulation of different environment and inducing factors [10]. TAM/M2 polarization enhanced the resistance of GC to DDP, leading to treatment failure [11]. Associated protein 1 enhanced tumor cells resistance to 5-fluorouracil through inducing TAM/M2 polarization [12]. Regulation of TAMs polarization may affect cancer progression and chemotherapy resistance. However, the relationship of TMEM205, TAMs polarization, and GC progression had not been reported.

In this study, we explored the effects of TMEM205 on the proliferation, stemness, migration, and EMT process of SGC-7901/DDP cells, clarified the molecular mechanism of TMEM205 inhibiting DDP-resistant GC cells by regulating TAMs polarization, and providing theoretical basis for clinical treatment of DDP-resistant GC.

## Materials and methods

### Cell lines

The human normal gastric epithelial cells (GES-1) and the human GC cells (SGC-7901) were obtained from the Cancer Research Center of Yanbian University. The DDP-resistant GC cells (SGC-7901/DDP) and THP-1 cells were purchased from Shanghai Fuheng company. All cells were cultured in RPMI-1640 medium containing 10% FBS and 1% penicillin–streptomycin and at 37 °C, 5% CO_2_.

### Transfection

We purchased TMEM205-specific siRNA from RIBOBIO (Guangzhou, China), including si-TMEM205-1, si-TMEM205-2, si-TMEM205-3, and siRNA (si-Con). The si-TMEM205-1, si-TMEM205-2, and si-TMEM205-3 sequences are GCTGTCCTCT CTTTGCAAT, TCGGACTAGTGCAGAGCAA, and TCTGCATCTTGGCTTCACA, respectively. 1 × buffer, siRNA and dye transfer solution were mixed and incubated at RT for 20 min, and added to penicillin–streptomycin-free RPMI-1640 cultured SGC-7901/DDP cells for 48 h.

### Established co-cultured system

Cells (1 × 10^5^/well) were inoculated in upper chamber of transwell device (BD Biosciences, Piscataway, NJ, USA). THP-1 cells were induced to macrophages and inoculated in lower chamber, and added 1.46 μg/mL DDP to the corresponding wells. After 48 h, THP-1 cells were detected by western blot, immunofluorescence, and flow cytometry assays.

### Western blot assay

Cells were lysed with appropriate RIPA buffer containing appropriate protease and phosphatase inhibitor (CoWin Biosciences, Beijing, China). Protein concentration was measured by BSA Protein Assay Kit (Roche, Basel, Switzerland). Denatured proteins were separated by SDS-PAGE glue and transferred to PVDF membranes (Millipore, Billerica, MA, USA). PVDF membranes were incubated with corresponding primary antibody overnight at 4 °C. On the next day, PVDF membranes were incubated with corresponding secondary antibody at RT for 1 h. The bands were obtained using the Gel Imaging System (Bio-Rad, Hercules, CA, USA). Names and item numbers of all the antibodies used in the experiment: Santa Cruz Biotechnology: CD44 (DF1485); sc-7297; Oct-4 (H-134) sc-9081; Nanog (1E6C4) sc-293121; Sox2 (E-4) sc-365823; iNOS (C-11) sc-7271; TMEM205 (B-5) sc-514568; E-cadherin (G-10): sc-8426; ZO-1 (R40.76) sc-33725; twist (Twist2C1a) sc-81417; N-cadherin (13A9) sc-59987; Vimentin (V9) sc-6260; SLUG (A-7) sc-166476; SNAI 1 (G-7) sc-271977; VEGF (JH121) sc-57496; MMP2 (8B4) sc-13595; MMP9 (E-11) sc-393859; Wnt-5a (A-5) sc-365370; beta-catenin (BDI870) sc-59896; c-Myc (A-14) sc-789; MDR1/ABCB1 (G-1) sc-13131; MRP1 (QCRL-1) sc-18835; caspase-3 (3CSP01) sc-65496; caspase-8 (3C121) sc-70501; caspase-9 (96.1.23) sc-56076; Bax (B-9) sc-7480; Bcl-2 (C-2) sc-7382. Cell Signaling Technology: Oct-4 Antibody #2750; CD206/MRC1 (E6T5J) XP Rabbit mAb #24,595; CD86 (E5W6H) Rabbit mAb #19,589); 3195 E-Cadherin (24E10) Rabbit mAb.

### MTT assay

SGC-7901 cells (1 × 10^3^/well) were inoculated in 96-well plates, and treated with different concentration of DDP (0, 0.4, 0.8, 1.2, 1.6, 2 μg/mL) for 48 h. SGC-7901/DDP cells (1 × 10^3^/well) were inoculated in 96-well plates, and treated with different concentration of DDP (0, 0.5, 1.0, 2.0, 4.0, 8.0 μg/mL) for 48 h. 100 μL MTT (1 mg/mL) was added to each well for 4 h at 37 °C. Absorbance value was measured at 490 nm after adding 100 μL DMSO to each well.

### Clone-formation assay

Cells (500/well) were inoculated in 6-well plates for 14 days. Cells were fixed with 4% paraformaldehyde for 30 min, washed with PBS, and stained with hematoxylin at RT. The number of colony formation was analyzed.

### EDU assay

SGC-7901/DDP cells (5 × 10^4^/well) were inoculated in 96-well plates, and corresponding wells were treated with 1.46 μg/mL DDP for 48 h. 100 μL EDU medium was added to each well for 2 h at 37 °C. 4% paraformaldehyde fixed cells for 30 min. 50 μL glycine (2 mg/mL) incubated cells for 5 min. 100 μL 0.5% Triton X-100 (CWBIO Biosciences) permeabilizing agent incubated cells for 5 min. Apollo staining reaction solution incubated cells at RT for 30 min, permeabilizing agent to decolorize cells and methanol to wash cells. 1 × Hoechst33342 reaction solution incubated cells for 30 min at RT, followed by using inverted microscope (NIH, Bethesda, MD, USA) to take pictures.

### Wound-healing assay

SGC-7901/DDP cells were inoculated in 6-well plates. The cells were scratched with 200 μL tips until fused to 80%. 1.46 μg/mL DDP was added into corresponding wells. The images were obtained at 0 h, 24 h and 48 h using an inverted microscope.

### Transwell assay

SGC-7901/DDP cells (1 × 10^5^/well) were inoculated into upper chamber of transwell device (BD Biosciences, Piscataway, NJ, USA), and RPMI-1640 complete medium was added into the lower chamber. 1.46 μg/mL DDP was added to corresponding wells. Cells were fixed with 4% paraformaldehyde and stained with hematoxylin after 48 h. Cells were counted under a microscope.

### Immunofluorescence (IF) assay

Cells were inoculated in 6-well plates. 1.46 μg/mL DDP was added to corresponding wells and cultured for 48 h at 37 °C. Cells were fixed with 4% paraformaldehyde and permeabilized with 0.5% Triton X-100. Cells were blocked with 3% BSA (Solarbio, Beijing, China) for 2 h at RT. Primary antibodies incubated cells at 4 °C overnight. Primary antibodies include E-cadherin (1:200), Vimentin (1:100), β-catenin (1:100), CD44 (1:100) MDR1 (1:100) MRP1 (1:100) (Santa Cruz Biotechnology, Dallas, TX, USA); CD86 (1:100), CD206 (1:100), Oct4 (1:100) (Cell Signaling Technology, USA). On the next day, cells were incubated with secondary antibodies for 2 h at RT, then counterstained with DAPI (Solarbio, Beijing, China). Finally, this assay used LeicaSP5II confocal microscope for imaging.

### Endothelial tube formation assay

50 μL of Matrigel (BD Biosciences, Piscataway, NJ, USA) mixture (serum-free RPMI-1640 medium: Matrigel = 1:1) was added into 96-well plate, and put it at 37 °C until Matrigel was completely solidified. HUVECs (1 × 10^5^/well) were inoculated into 96-well plates containing Matrigel. Lastly, this assay used microscope to collect microtubule formation pictures after 6 h.

### Flow cytometry assay

THP-1 cells (5 × 10^5^/tube) were re-suspended in the cell staining buffer after inducting to macrophages. THP-1 cells were stained with APC-CD86, (Biolegend, San Diego, USA) for 20 min at 4 °C. The cells were washed with intracellular staining buffer, and added intracellular fluorescent staining with FITC-68 and PE-CD206, (Biolegend, San Diego, USA) for 20 min at 4 °C. 500 μL cell staining buffer was added to re-suspend the cells, and then analyzed with the BD Accuri flow cytometer (BD Bioscience, Piscataway, NJ, USA).

### Database assay

We used The Human Protein Atlas database (http://www.proteinatlas.org/), Ualcan database (http://ualcan.path.uab.edu), CCLE database (https://portals.broadinstitute. org/ccle) and TIMER database (http://timer.comp-genomics.org) to analyze the expression of TMEM205 in cancer.

### In vivo tumorigenesis assay

This study utilized 20 male BALB/c nude mice (5 weeks old) purchased from Beijing Vital River Laboratory Animal Technology Co., Ltd. The mice were bred in a pathogen-free environment at a temperature of 22 °C, humidity of 50%, and a 12-h light/dark cycle. Subcutaneous xenograft tumor models were established by subcutaneously injecting a mixture of 5 × 10^6^ SGC-7901/DDP cells and Matrigel (BD Biosciences, Franklin Lakes, NJ, USA) into the right inguinal area of 10 nude mice. Subsequently, another set of 10 nude mice received subcutaneous injections of a mixture of 5 × 10^6^ SGC-7901/DDP (si-TMEM205-2) cells and Matrigel into the right inguinal area to establish subcutaneous xenograft tumor models. When the average tumor volume of the tumor-bearing mice reached 150–200 mm^3^, the mice were divided into four groups (control group n = 5, si-TMEM205-2 group n = 5, DDP group n = 5, si-TMEM205-2 + DDP group n = 5). The xenograft mice in the control group and si-TMEM205-2 group received physiological saline treatment, while the DDP group and si-TMEM205-2 + DDP group received cisplatin (10 ug) treatment via intraperitoneal injection every 3 days for a total of 7 injections. Tumor volume was measured using the modified ellipsoid formula (volume = 1/2[length × width^2^]). After 28 days of treatment with physiological saline or DDP, the mice were euthanized. Tumor tissues, as well as liver, kidney, and spleen tissues, were collected. The tissues were fixed in 10% formalin, embedded in paraffin for further hematoxylin and eosin (HE) staining or immunohistochemical analysis (Santa Cruz Biotechnology: Ki67 (Ki-67) sc-23900, CD44 (DF1485); E-cadherin (G-10); Vimentin (V9) sc-6260; MDR1/ABCB1 (G-1) sc-13131; MRP1 (QCRL-1) sc-18835); (Cell Signaling Technology: CD206/MRC1 (E6T5J) XP Rabbit mAb #24,595; Cell Signaling Technology: CD86 (E5W6H) Rabbit mAb #19,589). All experiments were approved by the Yanbian University Animal Ethics Committee.

### Immunohistochemistry staining

The tumor tissue was placed in 4% paraformaldehyde for 24 h to facilitate fixation. Subsequently, transfer appropriately sized tissue samples into embedding boxes for dehydration and embedding. The microtome was activated, securing the pre-cooled wax block in the refrigerator at 4 degrees. The blade's distance was adjusted from the wax block and set the slice thickness to 4 μm before initiating the slicing process. Tissue sections were deparaffinized and rehydrated for antigenic thermal repair, allowing natural cooling at room temperature. Endogenous peroxidase activity was countered by incubating sections in 3% hydrogen peroxide (H2O2) (ZSGB-BIO) for 30 min. Tissue sections were incubated overnight at 4 °C with the respective primary antibodies (CD206, CD86, E-Cadherin, Vimentin, Ki67, CD44). On the following day, wet cassettes were prewarmed at 37 °C for 1 h, and then immersed sections in a PBS rinse within a small dye vat. Sections were incubated with secondary antibodies, diluted with PBS (protected from light), in a molecular hybridization chamber at 37 °C for 1 h. Sections were washed with PBS three times for 5 min each. Successively stain tissue sections with 3,3′-diaminobenzidine (DAB) (ZSGB-BIO) and hematoxylin solution**.**

### HE staining

After paraffin embedding and dehydration, tissue sections are washed with PBS. The sections are then immersed in hematoxylin dye, which binds to the nuclear DNA, presenting a purple color. Subsequently, the sections are immersed in eosin dye, which stains the cytoplasm, presenting a pink color. Following alcohol and clearing agent treatment, the sections are dehydrated and cleared. Utilizing a neutral resin mounting medium, the slides are sealed to protect the sections and enhance durability. Ultimately, observation of the HE-stained sections under a microscope allows for a clear visualization of tissue architecture and cell morphology.

### Statistical assay

GraphPad Prism 8.0 software (GraphPad, La Jolla, CA, USA) was used to analyze data. Two groups of data were compared by Student’s *t* test. Multiple groups of data were compared by one-way ANOVA. *P* value < 0.05 was statistically significant (*P < 0.05, **P < 0.01, ***P < 0.001, ****P < 0.0001), *P* value > 0.05 has no statistically significant (ns). All experiments were repeated three times.

## Results

### DDP-resistant GC cells promote cancer stemness

**Fig. 1:**
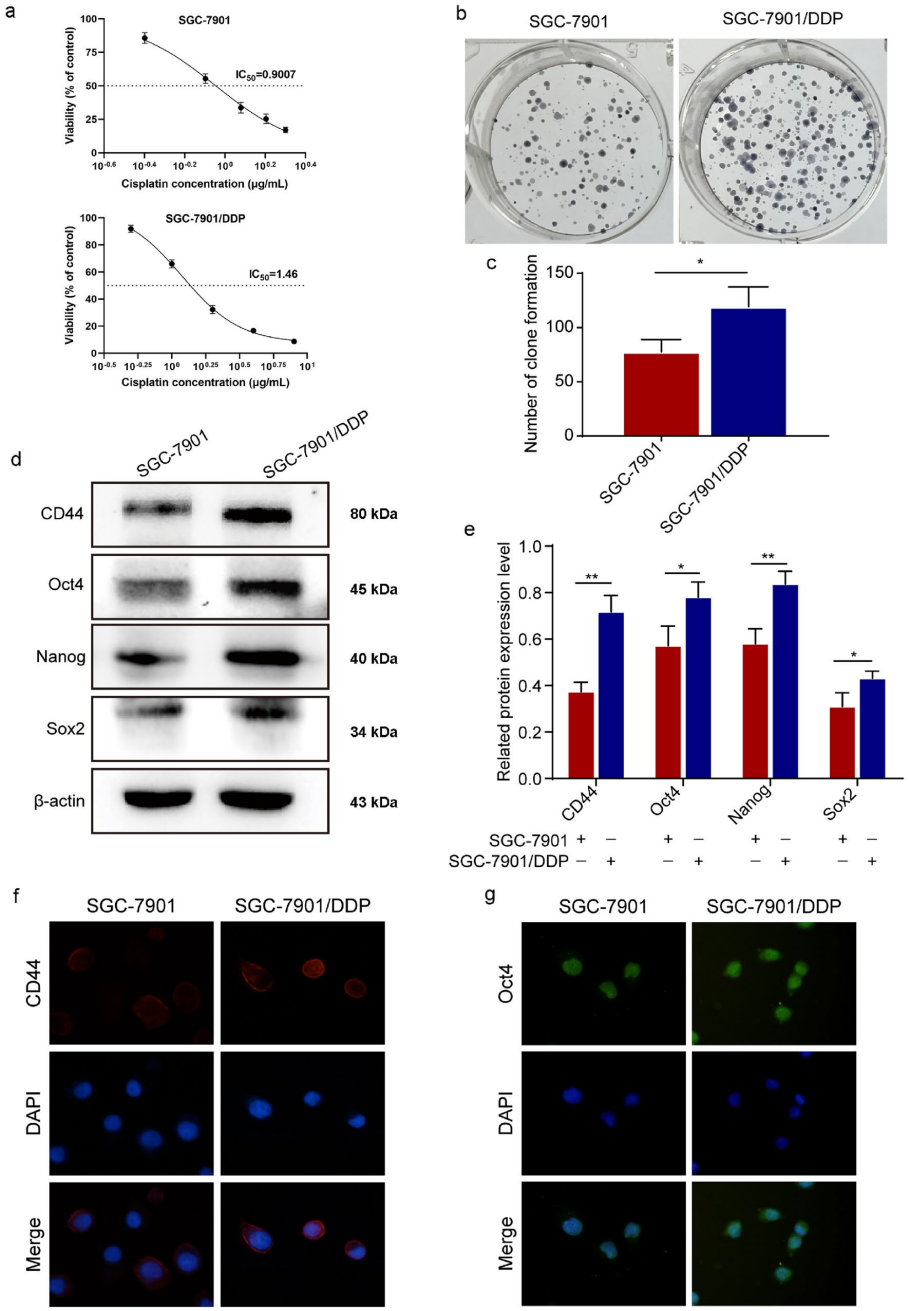
DDP-resistant GC cells promote cancer stemness. **a** MTT assay detected the effects of DDP on the proliferation of SGC-7901 and SGC-7901/DDP cells. **b–c** Colony-formation assay detected the proliferation of SGC-7901 and SGC-7901/DDP cells. **d–e** Western blot assay showed the expression of CD44, Oct4, Nanog, and Sox2 in SGC-7901 and SGC-7901/DDP cells. **f, g **IF detected the fluorescence intensity of CD44 and Oct4 in SGC-7901 and SGC-7901/DDP cells (400 ×)

We detected the sensitivity of SGC-7901/DDP cells to DDP, and treated SGC-7901 and SGC-7901/DDP cells with different concentrations of DDP for 48 h. MTT assay showed that the IC_50_ value of SGC-7901 cells is 0.9 μg/mL, and the IC_50_ value of SGC-7901/DDP cells is 1.46 μg/mL (Fig. 1a). SGC-7901/DDP cells were more resistant to DDP compared with SGC-7901 cells. The colony-formation ability of SGC-7901/DDP cells was enhanced compared with SGC-7901 cells (Fig. 1b, c). Related studies showed that migration, invasion, and EMT progression of SGC-7901/DDP cells were enhanced compared with SGC-7901 cells [13–15]. In addition, we examined the effect of DDP-resistant GC on cancer stemness. Western blot assay showed that the stemness-related markers CD44, Oct4, Nanog, and Sox2 were up-regulated in SGC-7901/DDP cells compared with SGC-7901 cells (Fig. 1d, e). IF assay showed that the fluorescence intensity of CD44 and Oct4 was enhanced in SGC-7901/DDP cells compared with SGC-7901 cells (Fig. 1f, g).

### DDP-resistant GC cells promote TAM/M2 polarization

**Fig. 2:**
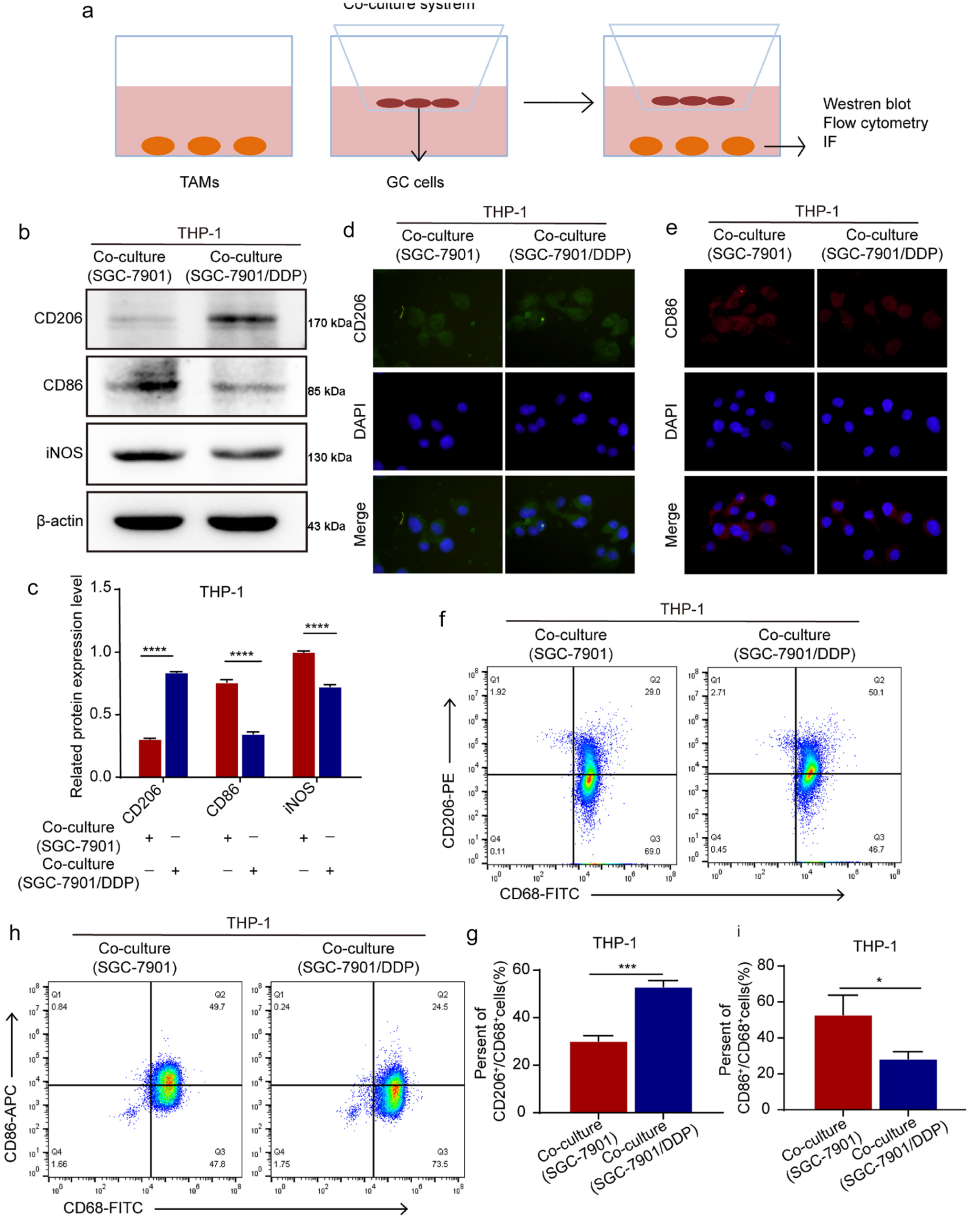
DDP-resistant GC cells induce TAM/M2 polarization. **a** Schematic diagram of co-culture system. **b–c** Western blot assay detected the expression of CD206, CD86, and iNOS in THP-1 cells of co-culture system. **d, e** IF detected the fluorescence intensity of CD206 and CD86 in THP-1 cells (400 ×). **f–i** Flow cytometry assay detected the percent of CD206^+^/CD68^+^cells and CD86^+^/CD68^+^cells in THP-1 cells

TAMs were associated with poor prognosis of cancer and involved in tumor angiogenesis and promoted tumor metastasis [16]. We detected the effect of DDP-resistant GC on TAM/M2 polarization in THP-1 cells of co-culture system (Fig. 2a). Western blot assay showed that SGC-7901/DDP cells up-regulated the expression of TAM/M2 marker CD206, and down-regulated the expression of TAM/M1 marker CD86 and iNOS in THP-1 cells compared with SGC-7901 cells (Fig. 2b, c). IF assay showed that SGC-7901/DDP cells enhanced the fluorescence intensity of CD206 and weakened the fluorescence intensity of CD86 in THP-1 cells compared with SGC-7901 cells (Fig. 2d, e). Flow cytometry assay showed that SGC-7901/DDP cells increased the percent of CD206^+^/CD68^+^ cells, and decreased the percent of CD86^+^/CD68^+^ cells in THP-1 cells compared with SGC-7901 cells (Fig. 2f–i). These results suggest that DDP-resistant in GC cells induced TAM/M2 polarization.

### TMEM205 enhances the proliferation and stemness of SGC-7901/DDP cells

**Fig. 3:**
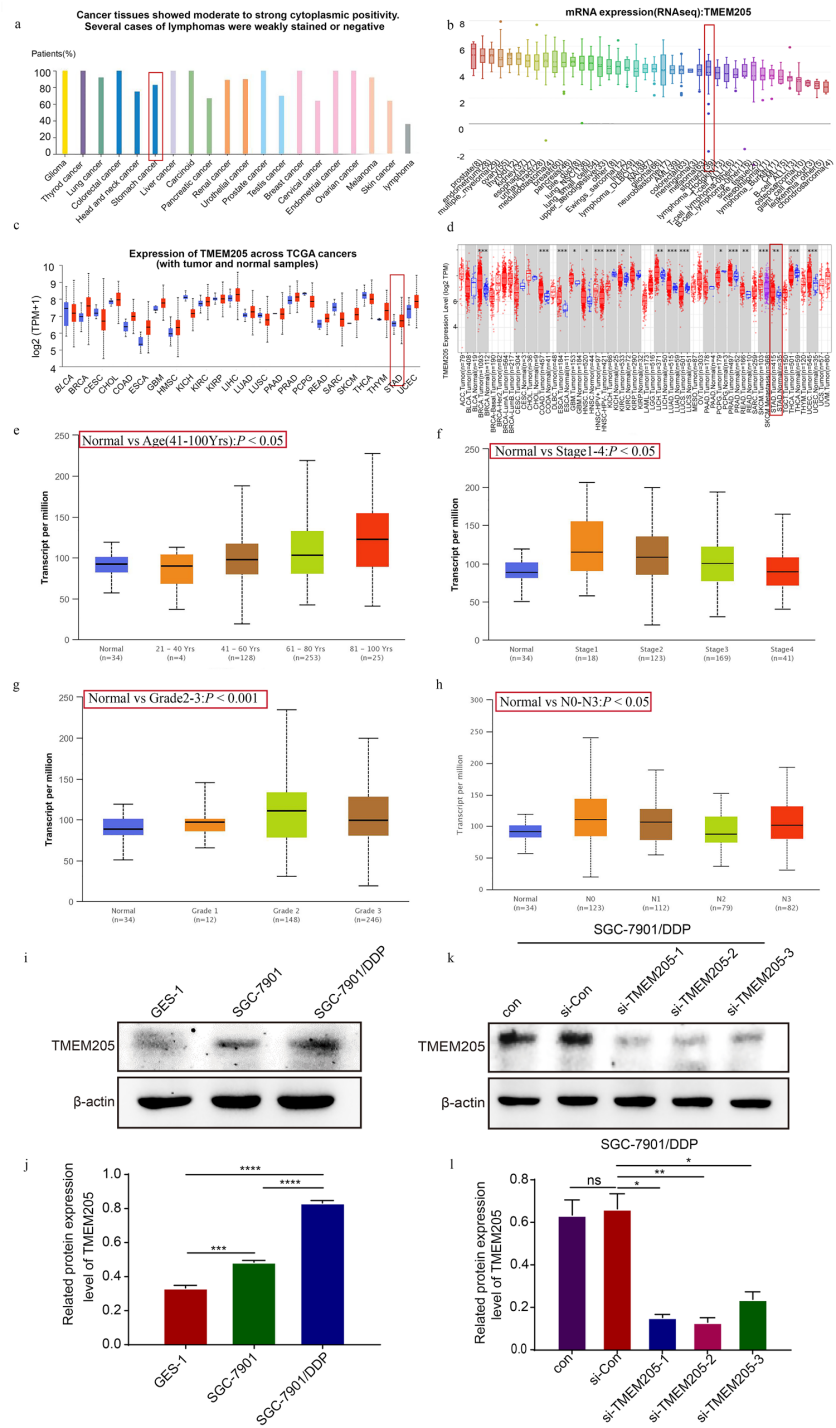
TMEM205 overexpressed in GC. **a–d** Ualcan, CCLE and TIMER databases detected the expression of TMEM205. **e–h** Ualcan database detected the relationship between TMEM205 and patient age, clinical stage, pathological grade, and lymph node metastasis of GC. **i-j** Western blot assay detected the expression of TMEM205 in GES-1, SGC-7901, and SGC-7901/DDP cells. **k-l** Western blot detected the expression of TMEM205 in si-TMEM205-transfected SGC-7901/DDP cells

We found that TMEM205 overexpressed in GC through The Human Protein Atlas, Ualcan, CCLE, and TIMER databases (Fig. 3a–d). Ualcan database showed that TMEM205 expression was correlated with age, clinical stage of GC, pathological grade of GC, and lymph node metastasis of GC (Fig. 3e–h). The above indicates that TMEM205 is involved in the malignant evolution of gastric cancer.

Then we explored the expression of TMEM205 in GC cells. Western blot assay showed that TMEM205 overexpressed in SGC-7901/DDP cells compared with GES-1 cells and SGC-7901 cells (Fig. 3i, j). Then SGC-7901/DDP cells were transfected using si-Con and three different TMEM205-specific siRNA. Western blot assay showed that si-TMEM205-2 had the most obvious knockdown effect and was selected for the subsequent assays (Fig. 3k–l). (Note: we performed the same experiment with another si-RNA (si-TMEM205-1) for fear of off-target effects. The results are shown in Supplementary Figs. 2, 3).

**Fig. 4:**
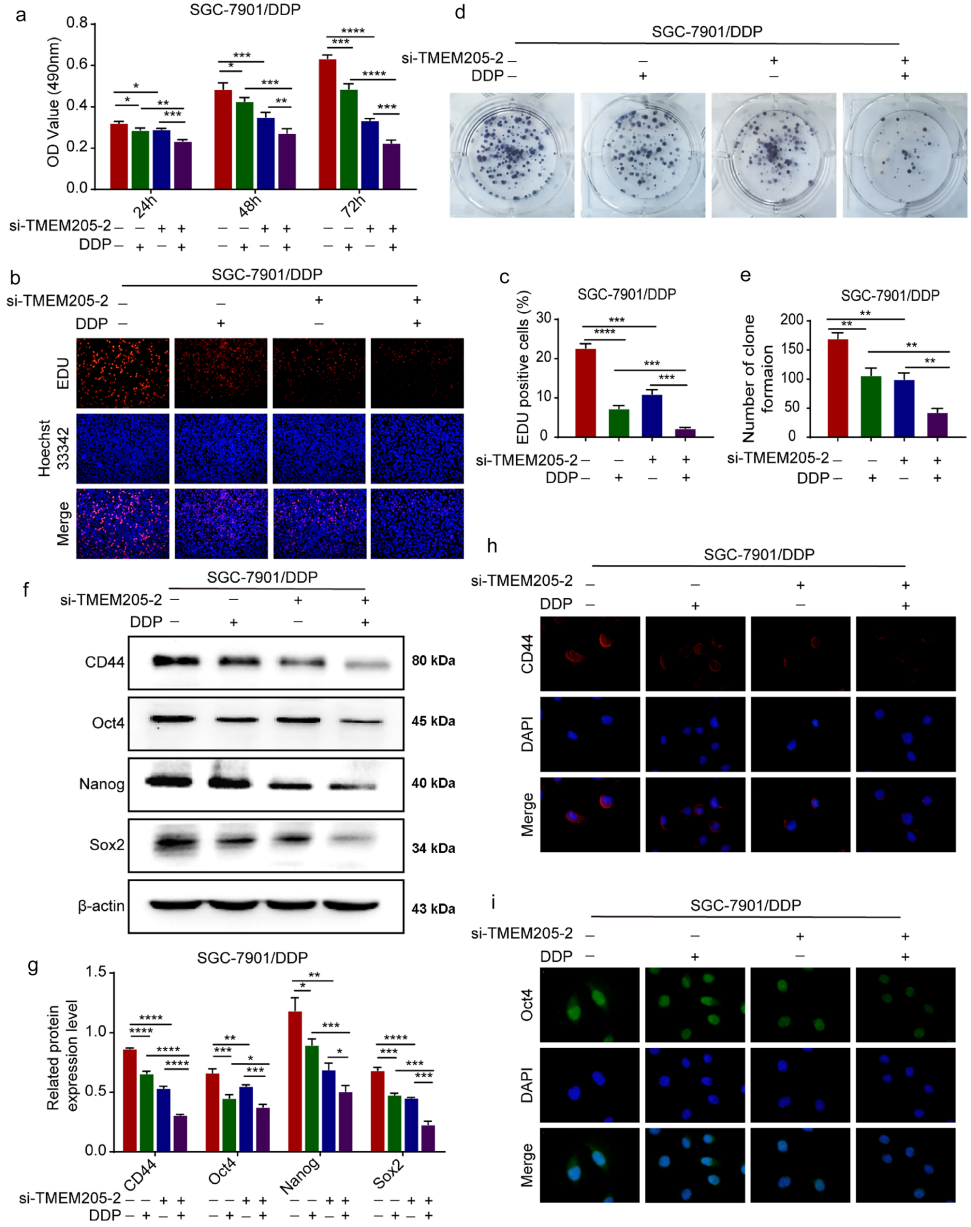
TMEM205 enhances the proliferation and stemness of SGC-7901/DDP cells. **a** MTT detected the proliferation of SGC-7901/DDP cells after using si-TMEM205-2 and/or DDP (1.46 μg/mL). **b, c** EDU assay detected the proliferation of SGC-7901/DDP cells of single or combined treatment groups. **d, e** Colony-formation assay detected the proliferation of SGC-7901/DDP cells of single or combined treatment groups. **f, g** Western blot assay detected the expression of stemness marker (CD44, Oct4, Nanog and Sox2) in SGC-7901/DDP cells of single or combined treatment groups. **h, i** IF assay detected the fluorescence intensity of CD44 and Oct4 of SGC-7901/DDP cells of single or combined treatment groups (400 ×)

We used si-TMEM205-2 and/or DDP (1.46 μg/mL) to detect the effect of TMEM205 on SGC-7901/DDP cell proliferation. MTT, EDU, and clone-formation assays showed that the combined treatment group significantly inhibited the proliferation of SGC-7901/DDP cells compared with the single treatment group (Fig. 4a–e). Western blot assay showed that the combined treatment group significantly down-regulated the expression of CD44, Oct4, Nanog, and Sox2 compared with the single treatment group (Fig. 4f, g). IF assay showed that the combined treatment group significantly weakened the fluorescence intensity of CD44 and Oct4 in SGC-7901/DDP cells compared with the single treatment group (Fig. 4h, i).

### TMEM205 promotes migration and EMT process of SGC-7901/DDP cells

**Fig. 5:**
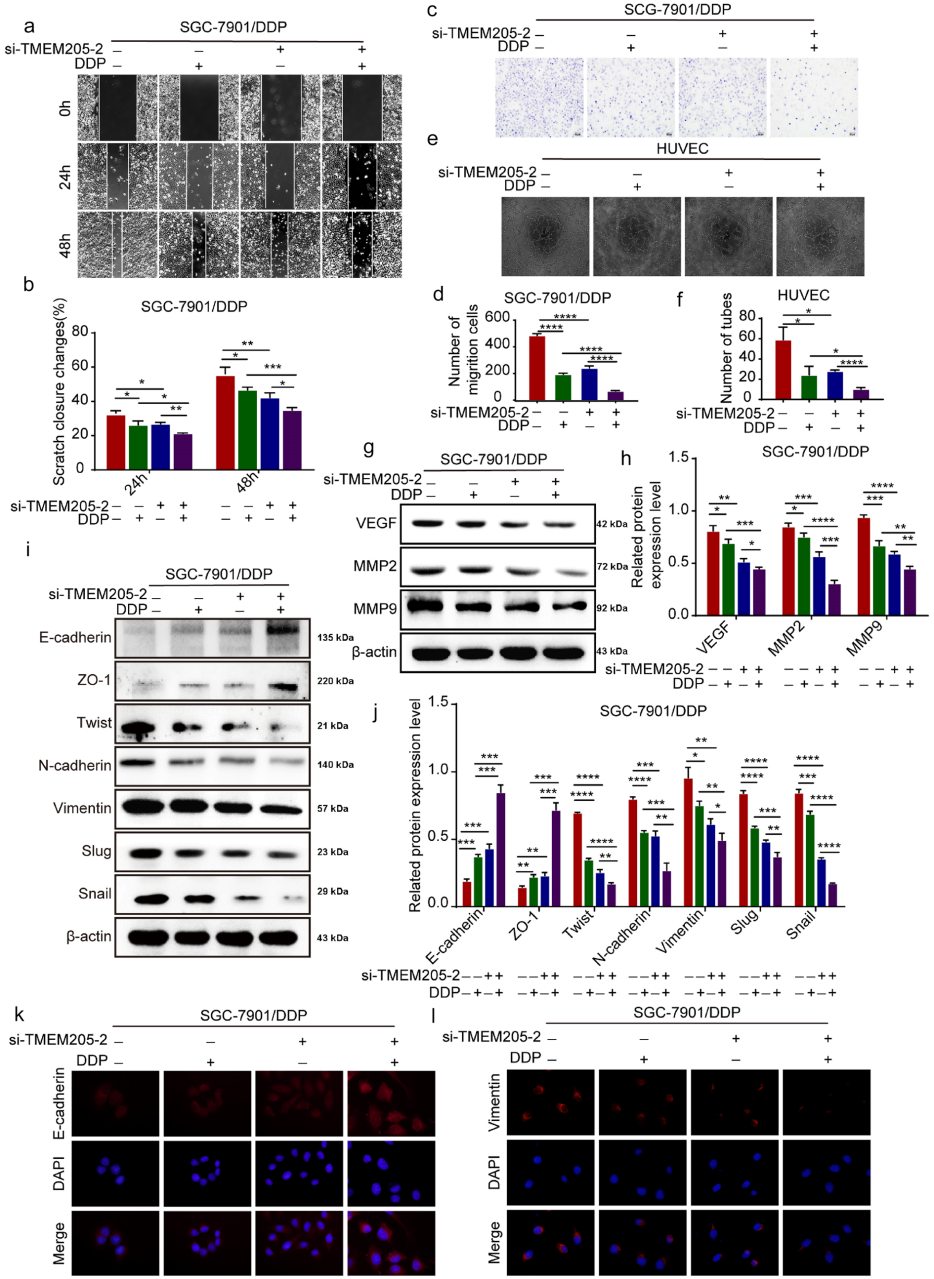
TMEM205 promotes migration and EMT process of SGC-7901/DDP cells. **a, b** Wound-healing assay detected the wound-healing capacity of SGC-7901/DDP cells of single or combined treatment groups. **c, d** Transwell assay detected the migration capacity of SGC-7901/DDP cells of single or combined treatment groups (200 ×). **e, f** Endothelial tube formation assay detected the microtubule formation of HUVECs of single or combined treatment groups (100 ×). **g, h** Western blot assay detected that the expression of VEGF, MMP2, and MMP9 in SGC-7901/DDP cells of single or combined treatment groups. **i, j** Western blot assay detected the expression of EMT-related markers of SGC-7901/DDP cells of single or combined treatment groups. **k, l** IF assay detected the fluorescence intensity of E-cadherin and Vimentin of SGC-7901/DDP cells of single or combined treatment groups (400 ×)

Metastasis was the main cause of poor prognosis of cancer, and angiogenesis was a necessary process for aggressive tumor growth and metastasis [17]. Then we detected the effects of TMEM205 on SGC-7901/DDP cells migration. Wound-healing and transwell assays showed that the combined treatment group significantly inhibited the migration of SGC-7901/DDP cells compared with the single treatment group (Fig. 5a–d). Endothelial tube formation assay showed that the combined treatment group significantly inhibited the microtubule formation of HUVECs compared with the single treatment group (Fig. 5e, f). Western blot assay showed that the combined treatment group significantly down-regulated the expression of VEGF, MMP2 and MMP9 in SGC-7901/DDP cells compared with the single treatment group (Fig. 5g, h).

The EMT process played important roles in tumor invasion, migration, and angiogenesis [18]. Therefore, we detected the effect of TMEM205 on EMT process of SGC-7901/DDP cells. Western blot assay showed that the combined treatment group significantly up-regulated the expression of epithelial markers E-cadherin and ZO-1, and down-regulated the expression of mesenchymal markers Twist, N-cadherin, Vimentin, Slug, and Snail in SGC-7901/DDP cells compared with the single treatment group (Fig. 5i,.j). IF assay showed that the combined treatment group significantly enhanced the fluorescence intensity of E-cadherin and weakened the fluorescence intensity of Vimentin in SGC-7901/DDP cells compared with the single treatment group (Fig. 5k, l).

### TMEM205 induces TAM/M2 polarization

**Fig. 6:**
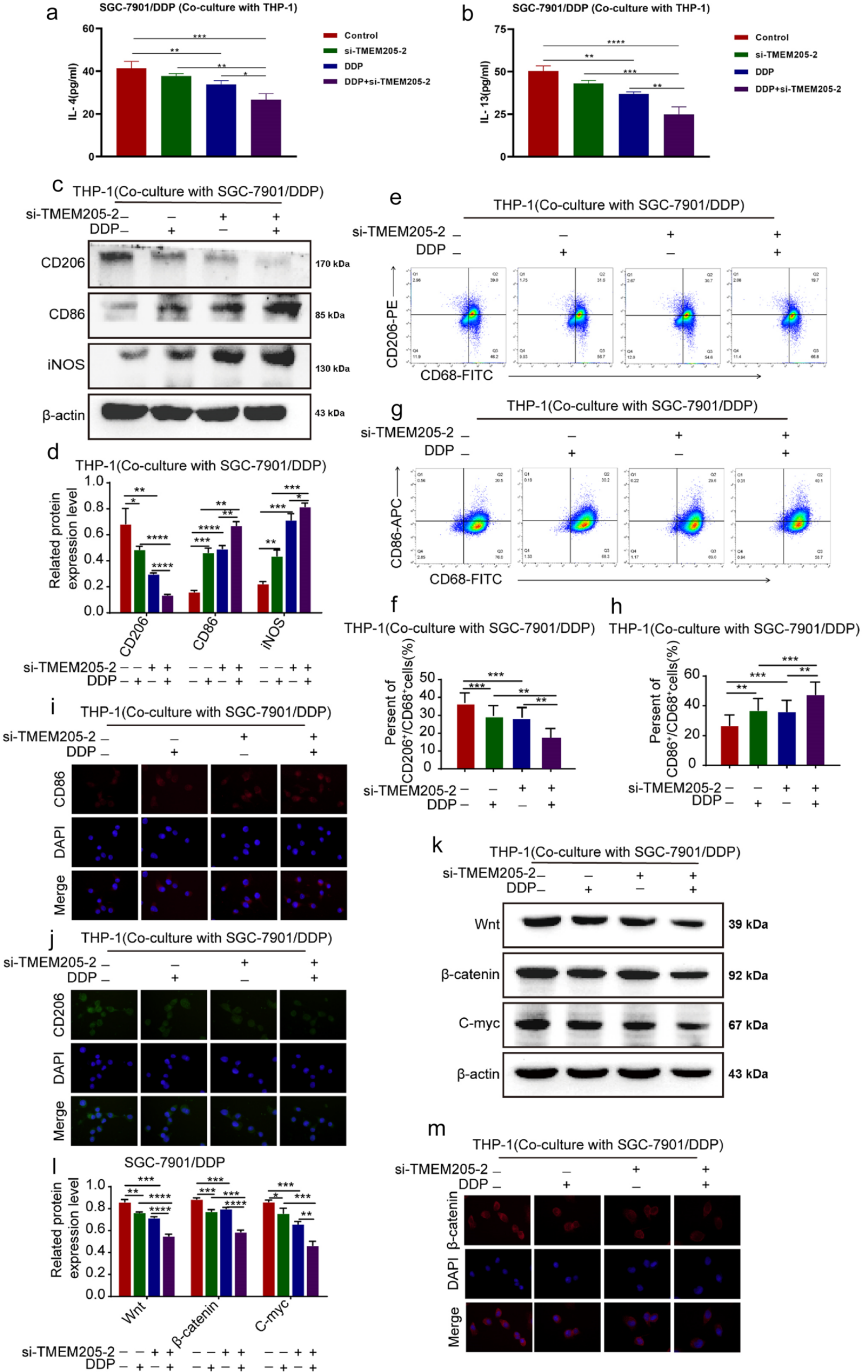
TMEM205 induces TAM/M2 polarization and activation of Wnt/β-catenin pathway in SGC-7901/DDP cells. **a, b** Expression levels of IL-4 and IL-13 in co-culture systems. **c, d** Western blot assay showed the expression of CD206 and CD86 in THP-1 cells of co-culture system of single or combined treatment groups. **e–h** Flow cytometry assay detected the percent of CD206^+^/CD68^+^cells and CD86^+^/CD68^+^cells in THP-1 cells. **i, j** IF assay detected the fluorescence intensity of CD206 and CD86 in THP-1 cells. **k, l** Western blot assay showed the expression of Wnt/β-catenin pathway-related proteins of SGC-7901/DDP cells of single or combined treatment groups. **m** IF detected the fluorescence intensity of β-catenin in SGC-7901/DDP cells of single or combined treatment groups

Macrophages can be specifically activated (polarized) by various biomolecules: endotoxin (M1), IFN-γ (M1), TNFα (M1), MCSF (M2), TGFβ (M2), IL-4 (M2), IL-10 (M2), IL-13 (M2), etc. [19, 20]. M2 macrophages are highly phagocytic and produce extracellular matrix (ECM), angiogenic factors, chemokines, and IL-10 [21–23]. IL-4, a potent M2a inducer, stimulates expression of several proteases with anti-parasitic properties. The binding of IL-4 to its receptor promotes nuclear translocation of STAT3/6, which activates M2-specific genes (Arg1, IL-10, etc.) in conjunction with other transcription factors (c-Myc, IRF4) [21]. In most studies, IL-4 and IL-13 are used to induce M2 polarization [24–26]. According to the above conclusion, we speculate that in the co-culture system of SGC-7901/DDP cells and THP-1 cells, multiple factors are involved in inducing TAM/M2 polarization. We extracted the supernatant of the co-culture system and measured the levels of IL-4 and IL-13 in the supernatant using ELISA kits (Shanghai Enzyme-linked Biotechnology; ml058093; ml063719). The results showed that compared to the control group, the levels of IL-4 and IL-13 in the si-TMEM205-2 group did not decrease significantly, but they significantly decreased in the DDP group and the combined group (Fig. 6a, b). The above results are consistent with the changes in M2 polarization observed in our study. Therefore, we believe that in the co-culture system, IL-4 and IL-13 induce M2 polarization.

We detected the effect of TMEM205 on the polarization of TAM/M2 in co-culture system. Western blot assay showed that the combined treatment group significantly down-regulated the expression of CD206 and up-regulated the expression of CD86 and iNOS in THP-1 cells compared with the single treatment group (Fig. 6c, d). Flow cytometry assay showed that the combined treatment group decreased the percent of CD206^+^/CD68^+^ cells and increased the percent of CD86^+^/CD68^+^ cells in THP-1 cells compared with the single treatment group (Fig. 6e–h). IF assay showed that the combined treatment group significantly weakened the fluorescence intensity of CD206 and enhanced the fluorescence intensity of CD86 in THP-1 cells compared with the single treatment group (Fig. 6i, j).

### TMEM205 induces activation of Wnt/β-catenin pathway in SGC-7901/DDP cells

The Wnt/β-catenin signaling pathway played active roles in promoting tumor formation, chemoresistance, and stemness [27, 28]. To detect the possible mechanism of TMEM205 in SGC-7901/DDP cells, we detected the effect of TMEM205 on Wnt/β-catenin signaling pathway. Western blot assay showed that the combined treatment group significantly down-regulated the expression of Wnt/β-catenin pathway-related proteins Wnt, β-catenin, and C-myc in SGC-7901/DDP cells compared with the single treatment group (Fig. 6k, l). IF assay showed that the combined treatment group inhibited the expression of β-catenin into nucleus of SGC-7901/DDP cells compared with the single treatment group (Fig. 6m).

### TMEM205 promotes cisplatin resistance in gastric *cancer*.

**Fig. 7:**
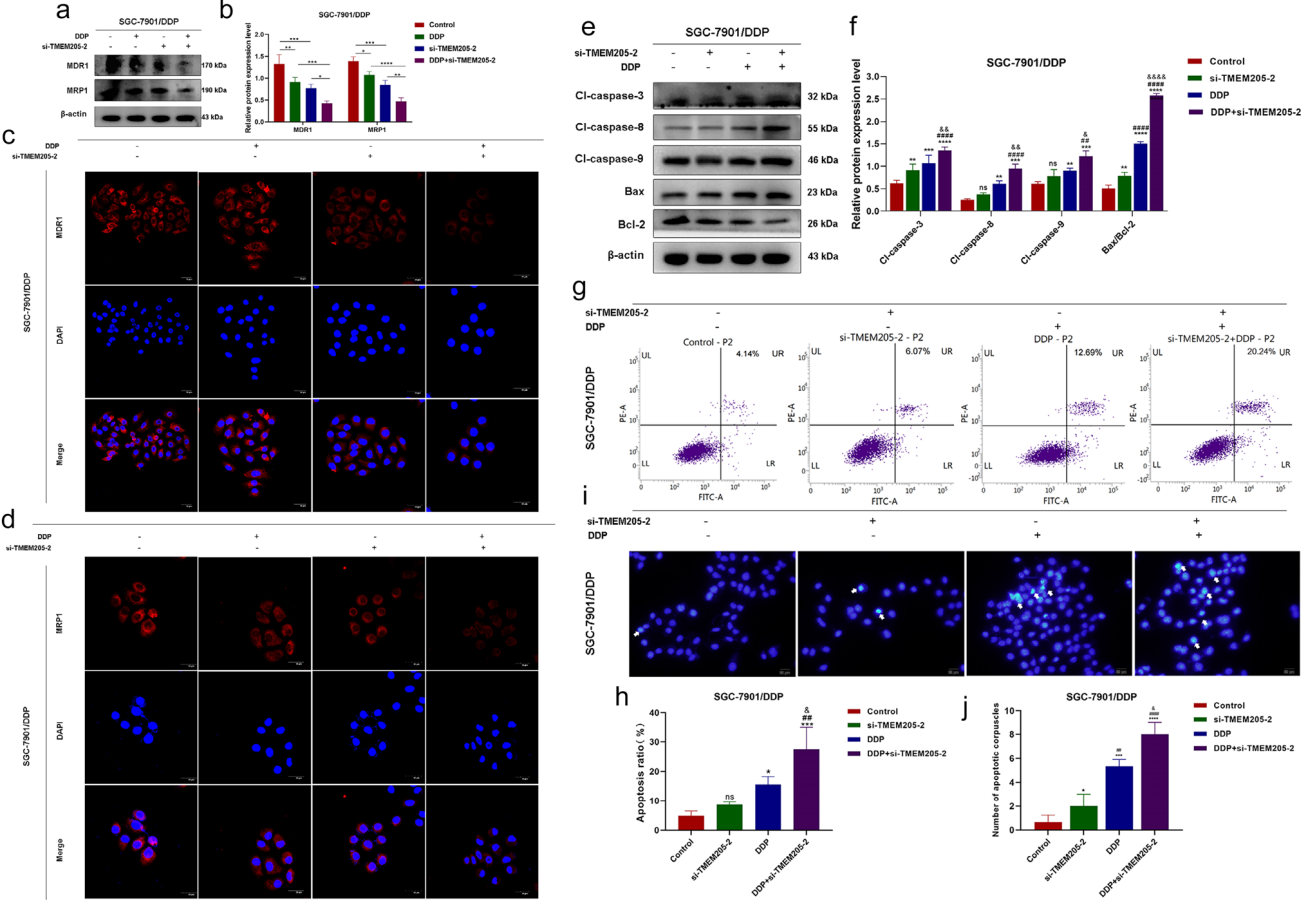
TMEM205 reverses cisplatin resistance in gastric cancer cells. **a–b** Western blot assay showing expression levels of drug-resistant proteins MDR1 and MRP1. **c–d** Immunofluorescence assay showing fluorescence intensity of drug-resistant proteins MDR1 and MRP1. **e–f** Expression levels of apoptosis-related proteins. **g–h** Flow cytometry to detect apoptosis rate in each group of cells. **i-j** The Hoechst 33,342 assay detects apoptotic vesicles in all groups

To elucidate the impact of TMEM205 on drug resistance in SGC-7901/DDP cells, we evaluated the expression levels of drug resistance-associated markers, specifically MDR1 and MRP1, through Western Blot and immunofluorescence techniques following diverse treatment regimens. The results obtained from the Western Blot analysis indicated a notable down-regulation of MDR1 and MRP1 expression in SGC-7901/DDP cells upon co-treatment with DDP and si-TMEM205-2, in contrast to si-TMEM205-2 or DDP alone (refer to Fig. 7a, b). Immunofluorescence experiments additionally demonstrated a significant reduction in the fluorescence intensity of MDR1 and MRP1 with the concurrent administration of si-TMEM205-2 and DDP (refer to Fig. 7c, d). These findings underscore the efficacy of TMEM205 suppression in alleviating drug resistance in SGC-7901/DDP cells.

Knockdown of TMEM205 reversed drug resistance in SGC-7901/DDP cells and increased their sensitivity to cisplatin. Therefore, we further validated the above results by apoptosis experiments. The Western Blot experiment results (Fig. 7e, f) show that compared to the control group, both the si-TMEM205-2 and DDP groups can upregulate apoptosis-related proteins and downregulate anti-apoptotic proteins in gastric cancer-resistant SGC-7901/DDP cells, with a more significant apoptotic effect observed in the combined treatment. To further investigate the impact of si-TMEM205-2 and DDP treatment on the apoptosis ability of SGC-7901/DDP cells, we used Annexin V-FITC/PI double staining to detect apoptosis under different treatment conditions. The experimental results (Fig. 7g, h) show that compared to the control group, there was no statistically significant increase in apoptosis rate in the si-TMEM205-2 group, but both the DDP and combination groups showed a statistically significant increase in apoptosis rate in SGC-7901/DDP cells. It is noteworthy that the combination treatment group exhibited a more significant increase in apoptosis rate. Finally, we observed the morphological changes in SGC-7901/DDP cells after different treatments using Hoechst 33,342 staining. The results (Fig. 7i, j) show that compared to the control group, the si-TMEM205-2 group, RG3 group, and combination treatment group all exhibited an increase in bright blue apoptotic bodies, with the combination treatment group showing the most significant increase. Additionally, typical morphological features of cell apoptosis, such as nuclear condensation and fragmentation, were observed in the combination treatment group.

The above results indicated that knockdown of TMEM205 increased the sensitivity of SGC-7901/DDP cells to cisplatin. si-TMEM205-2 combined with DDP promoted apoptosis in SGC-7901/DDP cells.

### TMEM205 promotes cisplatin resistance in gastric cancer by inducing TAM/M2 polarization in vivo

**Fig. 8:**
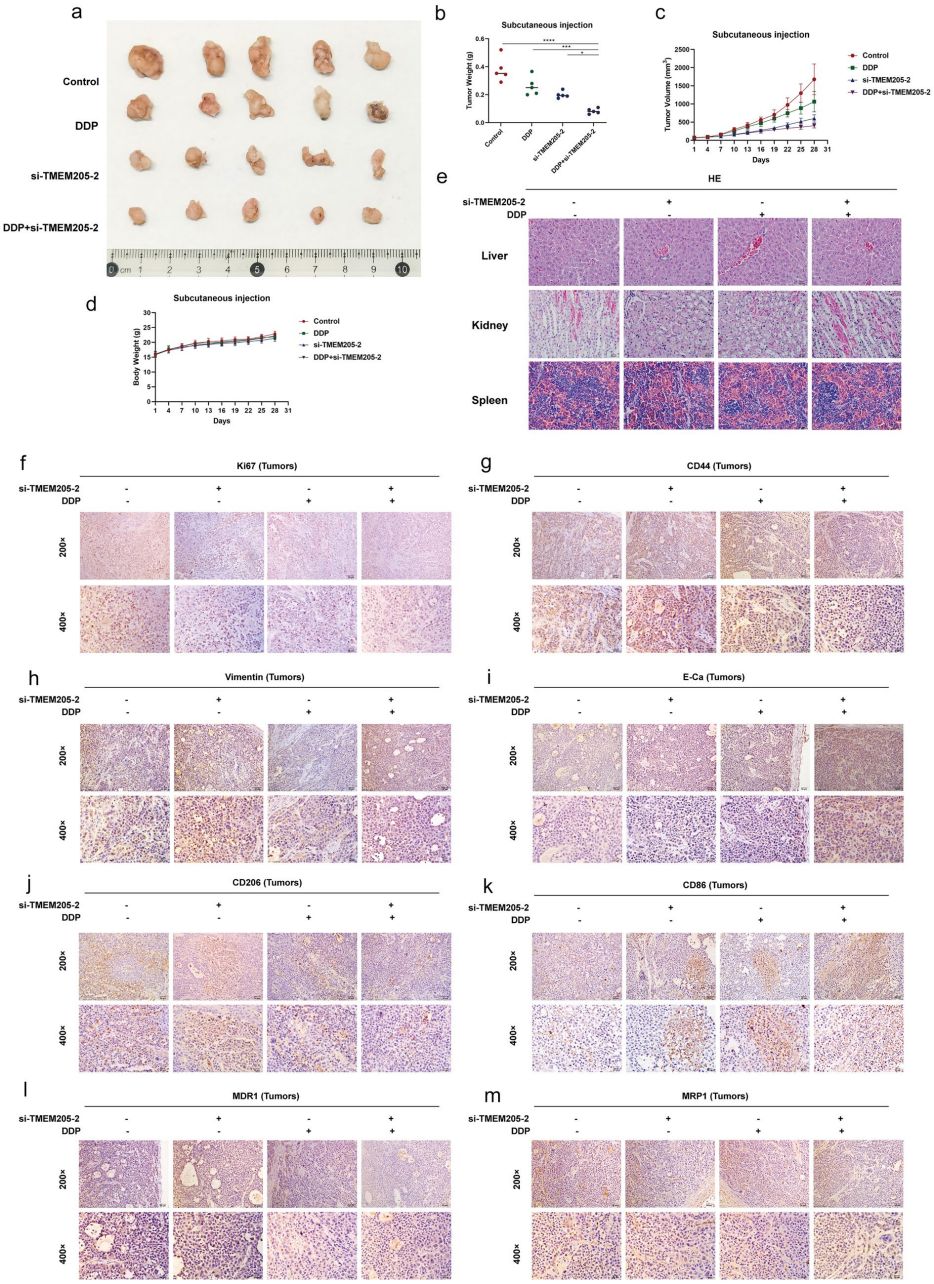
TMEM205 promotes cisplatin resistance in gastric cancer by inducing TAM/M2 polari-zation in vivo. **a–c** Tumor size, weight, and volume of subcutaneous injection; **d** body weight of mice by subcutaneous injection. **e **Hematoxylin and eosin (HE) staining of liver, kidney and spleen of mice after drug administration. **f–m** Expression of Ki67, CD44, E-cadherin, Vimentin, CD206, CD86, MDR1, and MRP1 in tumor tissues after subcutaneous injection detected by IHC

To further investigate the effect of TMEM205 on gastric cancer progression, we injected si-TMEM205-2-treated or untreated SGC-7901/DDP cells into thymus-free nude mice, respectively, and established a xenograft tumor model. The results showed that the combined effect of si-TMEM205-2 or DDP significantly inhibited the growth of gastric cancer tumors compared to si-TMEM205-2 or DDP alone (Fig. 8a–c). We detected the expression of EMT-related markers E-ca and Vimentin, TAMs/M2 marker CD206, TAMs/M1 marker CD86, cell proliferation-related marker Ki-67, and drug resistance-related markers MDR1 and MRP1 in the tumors of mice by immunohistochemistry, and got the same results with the experiments in vitro (Fig. 8f–m). These findings suggest that in the in vivo model, the combination of si-TMEM205-2 or DDP can significantly inhibit cisplatin-resistant gastric cancer development compared with si-TMEM205-2 or DDP alone. Notably, there were no significant differences in terms of body weight and normal tissue damage in the si-TMEM205-2 group, the DDP group, and the combination of the two groups compared with the control mice, suggesting that malignancy did not occur in the mice, and that the experiment was not toxic to other organs in the body of the mice (Fig. 8d, e). In the experiment, we utilized a cisplatin (DDP) concentration equivalent to the IC_50_ value (1.46 μg/mL). According to literature references [29–32], this concentration of cisplatin is deemed safe and effective for mice. As depicted in the HE staining images of liver, kidney, and spleen in Fig. 8e, comparison between the control group and the cisplatin group did not reveal any notable morphological changes indicative of hepatocyte deformation or other damages. Similarly, there were no apparent injuries observed in the kidney tubules or glomeruli, nor were there discernible signs of cell damage in the spleen. Upon comparing the control group with the si-TMEM205-2 group, we found no significant differences in liver, kidney, or spleen morphology, indicating no apparent damage in either group based on HE staining. Likewise, comparison between the cisplatin group and the DDP + si-TMEM205-2 group revealed no signs of hepatocyte damage or other abnormalities in the liver, kidney, or spleen. HE staining also did not demonstrate any notable differences between these two groups. Therefore, based on HE staining results, there were no evident differences in tissue morphology between the control and si-TMEM205-2 groups, nor between the cisplatin and DDP + si-TMEM205-2 groups.

These findings strongly suggest that TMEM205 is expected to be a potential therapeutic target for cisplatin-resistant gastric cancer.

## Discussion

As the common malignant tumor, chemotherapy was the important treatment for GC [15], and DDP was a more frequent choice of GC [2]. With the concentration of drugs increased, cancer cells gradually became resistant. Therefore, it is necessary to find a target that can reverse the drug resistance of GC to improve clinical benefit.

TMEMs were integral membrane proteins, which played important roles in the process of cell information transmission [3]. It has been shown that many TMEM proteins play their specific roles as oncogenes by promoting different processes, such as proliferation, migration, invasion, metastasis, and modulation, of the immune response of tumor cells [33]. Related studies have found that TMEM205 overexpressed in DDP-resistant ovarian cancer and participated in the therapeutic process of DDP for ovarian cancer [34]. TMEM88 overexpression inhibited cells proliferation, and enhanced the sensitivity of ovarian cancer to DDP [35]. These results indicated that TMEMs can affect the occurrence, development, and chemoresistance of cancer. However, whether TMEM205 could affect the sensitivity of GC to DDP had no studies. Our study results showed that TMEM205 enhanced the resistance of GC to DDP by promoting the proliferation and stemness of SGC-7901/DDP cells.

Metastasis is the act of cancer cells leaving the original location of the tumor and forming metastatic focus in a distant organ. This process is an important feature of cancer and constitutes the final step in malignant progression. It is also the leading cause of cancer-related death [36]. Metastasis can increase cancer resistance to chemotherapy drugs [37]. Meanwhile, metastasis involved multiple processes and interrelated steps, such as angiogenesis, EMT, and changes in the tumor microenvironment [38]. DDP-resistant lung cancer cells promoted distant metastasis of lung cancer [39]. TMEM230 promoted the malignant process of glioblastoma by promoting tumor angiogenesis [40]. It was shown that TMEM45B was overexpressed in samples from patients with clinical gastric cancer, and its depletion in cancer cell lines using RNA interference was found to be associated with inhibition of migratory and invasive behaviors. And it was observed that one of the main influencing factors was the alteration of the JAK2/STAT3 pathway that controls IL-6 secretion [41, 42]. Furthermore, in osteosarcoma cell lines, TMEM45B deletion was associated with reduced expression of β-catenin, a transcription factor that regulates EMT gene expression [43]. TMEM48 is localized to the nuclear membrane and is involved in the invasive process of lung cancer cell lines. In addition, MMP-2 and MMP-9 expression was reduced in TMEM48-silenced cells [44]. TMEM176A negatively regulates the invasive function of cells and it reduces the expression of MMP2, MMP9 in different types of tumors, such as glioma, colorectal, liver, and esophageal cancers [45, 46]. However, whether TMEM205 involved in cancer metastasis had no studies. Our results showed that TMEM205 promoted the migration of SGC-7901 cells, therapy accelerating the progression of GC.

EMT was the process by which epithelial cells acquire mesenchymal characteristics, and played important roles in the occurrence, invasion, metastasis, stemness and chemoresistance of cancer [47, 48]. Studies have shown that TMEM229 overexpression inhibited cells migration and invasion by preventing EMT process of lung cancer [49]. Connexin43 is a highly regulated intrinsic membrane protein containing four transmembrane domains, which include two extracellular loops, a cytoplasmic loop and cytoplasmic amino (-NH2) and carboxyl (-COOH) groups [50]. Connexin43 overexpression reversed tamoxifen resistance by inhibiting EMT process [51]. Whether TMEM205 affected the progression of GC through the EMT process had not been reported. Our results showed that TMEM205 promoted the proliferation, stemness, migration, and angiogenesis of SGC-7901/DDP cells by inducing the EMT process, thereby accelerating the malignant progress of GC.

TAMs involved in cancer proliferation, migration, invasion, and chemoresistance [52]. The phenotypic transition of TAMs contributed to cancer progression. Studies have found that TAMs could affect chemoresistance and cancer progression [53]. Triptolide affected DDP resistance in ovarian cancer by inducing TAM/M2 polarization [54]. However, whether TMEM205 affected the resistance of GC to DDP by regulating the polarization of TAMs had no studies. Our study results showed that TMEM205 enhanced the resistance of GC to DDP by inducing TAM/M2 polarization.

Activation of Wnt/β-catenin signaling pathway played important roles in tumor cell proliferation, stemness, migration, and chemotherapy resistance [27, 55]. The protein of β-catenin nuclear translocation could induce the activation of C-myc and CyclinD1, thereby activating the Wnt/β-catenin signaling pathway [56]. Studies have found that the competitive combination of TMEM88 and disheveled inhibited the activation of the Wnt/β-catenin pathway, thereby inhibiting the progression of triple-negative breast cancer [57]. It has been shown that some TMEM proteins are involved in tumor growth, proliferation, migration, and cell invasion through the Wnt/ b-catenin pathway, such as TMEM48, TMEM45B, and TMEM168, whereas TMEM170B and TMEM98 act as tumor development inhibitors through the same pathway. The exact mechanism by which TMEM proteins interact with the Wnt pathway is relatively straightforward for some TMEMs, while for others, it is completely unknown [58]. However, whether TMEM205 could affect cancer progression by regulating the Wnt/β-catenin pathway had not reported. Our study showed that TMEM205 promoted the proliferation, stemness, migration, angiogenesis, and EMT process of SGC-7901/DDP cells by activating Wnt/β-catenin.

Drug resistance is closely related to poor prognosis and cancer recurrence and is one of the main reasons for cancer treatment failure. The resistance to chemotherapy is not only related to the adaptation of tumor cells themselves, but also to the tumor microenvironment. After long-term treatment with chemotherapeutic agents, tumor cells may develop drug resistance [59]. It has been shown that TMEM45A is involved in the process of chemoresistance in breast and hepatocellular carcinoma; however, the mechanism of this protection remains unclear [60]. De Leon et al. validated the interaction of TMEM88 with the Wnt pathway in ovarian cancer. They investigated the link between this interaction and the observed chemoresistance. Overexpression of TMEM88 in drug-resistant cells inhibited the Wnt signaling pathway and decreased target gene expression, whereas activation of the Wnt pathway in drug-resistant cells increased cellular chemosensitivity to cisplatin. In addition, TMEM88 failure in cisplatin-resistant cells increased cell sensitivity to chemotherapeutic agents [35]. Our results showed that TMEM205 reduced the sensitivity of gastric cancer cells to cisplatin, which was closely associated with cisplatin resistance in gastric cancer cells, and silencing TMEM205 reversed cisplatin resistance in gastric cancer cells.

## Conclusion

The results of this study indicated that knocking down TMEM205 combined with DDP inhibited the proliferation, migration, angiogenesis, and EMT process of SGC-7901/DDP cells. In terms of mechanism, TMEM205 played an important role in DDP resistance and malignant progression of GC through Wnt/β-catenin signaling pathway. It is expected to be a potential therapeutic target for cisplatin-resistant gastric cancer. Research on TMEM205 is still in its infancy, and our study provides strong evidence that TMEM205 plays an important role in tumor progression and reversal of drug resistance. In the future, we will launch a series of studies around TMEM205 to unveil the various roles it plays in the tumor microenvironment.

### Supplementary Information

Below is the link to the electronic supplementary material.

Supplementary file1 (DOCX 795 KB)

## References

[1] Pawluczuk E, Łukaszewicz-Zając M, Mroczko B. The role of chemokines in the development of gastric cancer-diagnostic and therapeutic implications. Int J Mol Sci. 2020;21(22) 10.3390/ijms21228456 PMC7697532 33182840

[2] Smyth EC, Nilsson M, Grabsch HI, van Grieken NCT, Lordick F. Gastric cancer. Lancet. 2020;396(10251):635–48. 32861308 10.1016/S0140-6736(20)31288-5

[3] Schmit K, Michiels C. TMEM Proteins in Cancer: A Review. Front Pharmacol. 2018;9:1345. 30574087 10.3389/fphar.2018.01345 PMC6291505

[4] Saini U, Smith BQ, Dorayappan KDP, Yoo JY, Maxwell GL, Kaur B, et al. Targeting TMEM205 mediated drug resistance in ovarian clear cell carcinoma using oncolytic virus. J Ovarian Res. 2022;15(1):130. 36476493 10.1186/s13048-022-01054-5 PMC9730683

[5] Shen D-W, Ma J, Okabe M, Zhang G, Xia D, Gottesman MM. Elevated expression of TMEM205, a hypothetical membrane protein, is associated with cisplatin resistance. J Cell Physiol. 2010;225(3):822–8. 20589834 10.1002/jcp.22287 PMC2971691

[6] Shen DW, Gottesman MM. RAB8 enhances TMEM205-mediated cisplatin resistance. Pharm Res. 2012;29(3):643–50. 21969054 10.1007/s11095-011-0562-y PMC3288766

[7] Xu H, Dun S, Gao Y, Ming J, Hui L, Qiu X. TMEM107 inhibits EMT and invasion of NSCLC through regulating the Hedgehog pathway. Thorac Cancer. 2021;12(1):79–89. 33124203 10.1111/1759-7714.13715 PMC7779196

[8] Liu Y, Zheng Q, He G, Zhang M, Yan X, Yang Z, et al. Transmembrane protein 215 promotes angiogenesis by maintaining endothelial cell survival. J Cell Physiol. 2019;234(6):9525–34. 30370660 10.1002/jcp.27641 PMC6587792

[9] Yang B, Wang F, Zheng G. Transmembrane protein TMEM119 facilitates the stemness of breast cancer cells by activating Wnt/beta-catenin pathway. Bioengineered. 2021;12(1):4856–67. 34334123 10.1080/21655979.2021.1960464 PMC8806430

[10] Pan Y, Yu Y, Wang X, Zhang T. Tumor-associated macrophages in tumor immunity. Front Immunol. 2020;11: 583084. 33365025 10.3389/fimmu.2020.583084 PMC7751482

[11] Zheng P, Chen L, Yuan X, Luo Q, Liu Y, Xie G, et al. Exosomal transfer of tumor-associated macrophage-derived miR-21 confers cisplatin resistance in gastric cancer cells. J Exp Clin Cancer Res. 2017;36(1):53. 28407783 10.1186/s13046-017-0528-y PMC5390430

[12] He Z, Chen D, Wu J, Sui C, Deng X, Zhang P, et al. Yes associated protein 1 promotes resistance to 5-fluorouracil in gastric cancer by regulating GLUT3-dependent glycometabolism reprogramming of tumor-associated macrophages. Arch Biochem Biophys. 2021;702. 33727040 10.1016/j.abb.2021.108838

[13] Li P, Hu J, Shi B, Tie J. Baicalein enhanced cisplatin sensitivity of gastric cancer cells by inducing cell apoptosis and autophagy via Akt/mTOR and Nrf2/Keap 1 pathway. Biochem Biophys Res Commun. 2020;531(3):320–7. 32800561 10.1016/j.bbrc.2020.07.045

[14] Li C, Zou J, Zheng G, Chu J. MiR-30a Decreases Multidrug Resistance (MDR) of Gastric Cancer Cells. Med Sci Monit. 2016;22:4509–15. 27876712 10.12659/MSM.898415 PMC5123779

[15] Ge L, Li DS, Chen F, Feng JD, Li B, Wang TJ. TAZ overexpression is associated with epithelial-mesenchymal transition in cisplatin-resistant gastric cancer cells. Int J Oncol. 2017;51(1):307–15. 28534974 10.3892/ijo.2017.3998

[16] Cheng N, Bai X, Shu Y, Ahmad O, Shen P. Targeting tumor-associated macrophages as an antitumor strategy. Biochem Pharmacol. 2021;183: 114354. 33279498 10.1016/j.bcp.2020.114354

[17] Folkman JJ. Role of angiogenesis in tumor growth and metastasis. Semin Oncol. 2002;29(6 Suppl 16):15–8. 12516034 10.1053/sonc.2002.37263

[18] Yang F, Shao C, Wei K, Jing X, Qin Z, Shi Y, et al. miR-942 promotes tumor migration, invasion, and angiogenesis by regulating EMT via BARX2 in non-small-cell lung cancer. J Cell Physiol 2019; 234(12):23596–23607. 10.1002/jcp.28928 31236953

[19] Murray PJ, Allen JE, Biswas SK, Fisher EA, Gilroy DW, Goerdt S, et al. Macrophage activation and polarization: nomenclature and experimental guidelines. Immunity. 2014;41(1):14–20. 25035950 10.1016/j.immuni.2014.06.008 PMC4123412

[20] Morris DL, Singer K, Lumeng CN. Adipose tissue macrophages: phenotypic plasticity and diversity in lean and obese states. Curr Opin Clin Nutr Metab Care. 2011;14(4):341–6. 21587064 10.1097/MCO.0b013e328347970b PMC4690541

[21] Kiseleva V, Vishnyakova P, Elchaninov A, Fatkhudinov T, Sukhikh G. Biochemical and molecular inducers and modulators of M2 macrophage polarization in clinical perspective. Int Immunopharmacol. 2023;122:110583. 37423155 10.1016/j.intimp.2023.110583

[22] Fuentes L, Roszer T, Ricote M. Inflammatory mediators and insulin resistance in obesity: role of nuclear receptor signaling in macrophages. Mediators Inflamm. 2010;2010:219583. 20508742 10.1155/2010/219583 PMC2874923

[23] Bohlson SS, O’Conner SD, Hulsebus HJ, Ho M-M, Fraser DA. Complement, c1q, and c1q-related molecules regulate macrophage polarization. Front Immunol. 2014;5:402. 25191325 10.3389/fimmu.2014.00402 PMC4139736

[24] Scott TE, Lewis CV, Zhu M, Wang C, Samuel CS, Drummond GR, et al. IL-4 and IL-13 induce equivalent expression of traditional M2 markers and modulation of reactive oxygen species in human macrophages. Sci Rep. 2023;13(1):19589. 37949903 10.1038/s41598-023-46237-2 PMC10638413

[25] Lundahl MLE, Mitermite M, Ryan DG, Case S, Williams NC, Yang M, et al. Macrophage innate training induced by IL-4 and IL-13 activation enhances OXPHOS driven anti-mycobacterial responses. Elife. 2022. 10.7554/eLife.74690. 36173104 10.7554/eLife.74690 PMC9555863

[26] Cutolo M, Campitiello R, Gotelli E, Soldano S. The Role of M1/M2 Macrophage Polarization in Rheumatoid Arthritis Synovitis. Front Immunol. 2022;13:867260. 35663975 10.3389/fimmu.2022.867260 PMC9161083

[27] Zhang Y, Wang X. Targeting the Wnt/beta-catenin signaling pathway in cancer. J Hematol Oncol. 2020;13(1):165. 33276800 10.1186/s13045-020-00990-3 PMC7716495

[28] Xu X, Zhang M, Xu F, Jiang S. Wnt signaling in breast cancer: biological mechanisms, challenges and opportunities. Mol Cancer. 2020;19(1):165. 33234169 10.1186/s12943-020-01276-5 PMC7686704

[29] Guo X-F, Liu J-P, Ma S-Q, Zhang P, Sun W-D. Avicularin reversed multidrug-resistance in human gastric cancer through enhancing Bax and BOK expressions. Biomed Pharmacother. 2018;103:67–74. 29635130 10.1016/j.biopha.2018.03.110

[30] Ren X, Liu H, Zhang M, Wang M, Ma S. Co-expression of ING4 and P53 enhances hypopharyngeal cancer chemosensitivity to cisplatin in vivo. Mol Med Rep. 2016;14(3):2431–8. 27484725 10.3892/mmr.2016.5552 PMC4991689

[31] Peng L, Sang H, Wei S, Li Y, Jin D, Zhu X, et al. circCUL2 regulates gastric cancer malignant transformation and cisplatin resistance by modulating autophagy activation via miR-142-3p/ROCK2. Mol Cancer. 2020;19(1):156. 33153478 10.1186/s12943-020-01270-x PMC7643398

[32] Tang Q-F, Ji Q, Qiu Y-Y, Cao A-L, Chi Y-F, Liang B, et al. Synergistic Effect of Zuo Jin Wan on DDP-Induced Apoptosis in Human Gastric Cancer SGC-7901/DDP Cells. Evid Based Complement Alternat Med. 2014;2014:724764. 24723962 10.1155/2014/724764 PMC3958763

[33] Marx S, Dal Maso T, Chen JW, Bury M, Wouters J, Michiels C, et al. Transmembrane (TMEM) protein family members: Poorly characterized even if essential for the metastatic process. Semin Cancer Biol. 2020;60:96–106. 31454669 10.1016/j.semcancer.2019.08.018

[34] Calo CA, Smith BQ, Dorayappan KDP, Saini U, Lightfoot M, Wagner V, et al. Aberrant expression of TMEM205 signaling promotes platinum resistance in ovarian cancer: An implication for the antitumor potential of DAP compound. Gynecol Oncol. 2021. 10.1016/j.ygyno.2021.10.076. 34756749 10.1016/j.ygyno.2021.10.076

[35] de Leon M, Cardenas H, Vieth E, Emerson R, Segar M, Liu Y, et al. Transmembrane protein 88 (TMEM88) promoter hypomethylation is associated with platinum resistance in ovarian cancer. Gynecol Oncol. 2016;142(3):539–47. 27374141 10.1016/j.ygyno.2016.06.017 PMC4993677

[36] Gerstberger S, Jiang Q, Ganesh K. Metastasis. Cell. 2023;186(8):1564–79. 37059065 10.1016/j.cell.2023.03.003 PMC10511214

[37] Huang YK, Busuttil RA, Boussioutas A. The Role of Innate Immune Cells in Tumor Invasion and Metastasis. Cancers (Basel). 2021;13:23. 10.3390/cancers13235885 PMC8656477 34884995

[38] Guan X. Cancer metastases: challenges and opportunities. Acta Pharm Sin B. 2015;5(5):402–18. 26579471 10.1016/j.apsb.2015.07.005 PMC4629446

[39] Chen X, Lv Y, Xu K, Wang X, Xiang RJC. DCBLD2 Mediates epithelial-mesenchymal transition-induced metastasis by cisplatin in lung adenocarcinoma. Cancers. 2021;13(6):1403. 33808696 10.3390/cancers13061403 PMC8003509

[40] Cocola C, Magnaghi V, Abeni E, Pelucchi P, Martino V, Vilardo L, et al. Transmembrane Protein TMEM230, a Target of Glioblastoma Therapy. Front Cell Neurosci. 2021;15:703431. 34867197 10.3389/fncel.2021.703431 PMC8636015

[41] Shen K, Yu W, Yu Y, Liu X, Cui X. Knockdown of TMEM45B inhibits cell proliferation and invasion in gastric cancer. Biomed Pharmacother. 2018;104:576–81. 29803169 10.1016/j.biopha.2018.05.016

[42] Itoh H, Kadomatsu T, Tanoue H, Yugami M, Miyata K, Endo M, et al. TET2-dependent IL-6 induction mediated by the tumor microenvironment promotes tumor metastasis in osteosarcoma. Oncogene. 2018;37(22):2903–20. 29515232 10.1038/s41388-018-0160-0

[43] Li Y, Guo W, Liu S, Zhang B, Yu BB, Yang B, et al. Silencing Transmembrane Protein 45B (TNEM45B) Inhibits Proliferation, Invasion, and Tumorigenesis in Osteosarcoma Cells. Oncol Res. 2017;25(6):1021–6. 28244852 10.3727/096504016X14821477992177 PMC7841085

[44] Qiao W, Han Y, Jin W, Tian M, Chen P, Min J, et al. Overexpression and biological function of TMEM48 in non-small cell lung carcinoma. Tumour Biol. 2016;37(2):2575–86. 26392108 10.1007/s13277-015-4014-x

[45] Li H, Zhang M, Linghu E, Zhou F, Herman JG, Hu L, et al. Epigenetic silencing of TMEM176A activates ERK signaling in human hepatocellular carcinoma. Clin Epigenetics. 2018;10(1):137. 30400968 10.1186/s13148-018-0570-4 PMC6219251

[46] Wang Y, Ji L, Ji C, Wang F. Multi-omics approaches establishing histone modification based prognostic model in glioma patients and further verification of the carcinogenesis mechanism. Funct Integr Genomics. 2023;23(4):307. 37730879 10.1007/s10142-023-01229-3

[47] Pastushenko I, Blanpain C. EMT Transition States during Tumor Progression and Metastasis. Trends Cell Biol. 2019;29(3):212–26. 30594349 10.1016/j.tcb.2018.12.001

[48] Ashrafizadeh M, Zarrabi A, Hushmandi K, Kalantari M, Mohammadinejad R, Javaheri T, et al. Association of the Epithelial-Mesenchymal Transition (EMT) with Cisplatin Resistance. Int J Mol Sci. 2020;21:11. 10.3390/ijms21114002 PMC7312011 32503307

[49] Zhang X, He Y, Jiang Y, Bao Y, Chen Q, Xie D, et al. 2021 **TMEM229A suppresses nonsmall cell lung cancer progression via inactivating the ERK pathway**. *Oncol Rep.*; 46(2). 10.3892/or.2021.8127 PMC8261197 34184076

[50] Solan JL, Lampe PD. Connexin43 phosphorylation: structural changes and biological effects. Biochem J. 2009;419(2):261–72. 19309313 10.1042/BJ20082319 PMC2669545

[51] Wu DP, Zhou Y, Hou LX, Zhu XX, Yin XXJIjobs. Cx43 deficiency confers EMT-mediated tamoxifen resistance to breast cancer via c-Src/PI3K/Akt pathway. Int J Bio Sci. 2021. 10.7150/ijbs.55453. 10.7150/ijbs.55453 PMC8315014 34326682

[52] Boutilier AJ, Elsawa SF. Macrophage Polarization States in the Tumor Microenvironment. Int J Mol Sci. 2021;22(13):6995. 34209703 10.3390/ijms22136995 PMC8268869

[53] Qin T, Li B, Feng X, Fan S, Liu L, Liu D, et al. Abnormally elevated USP37 expression in breast cancer stem cells regulates stemness, epithelial-mesenchymal transition and cisplatin sensitivity. J Exp Clin Cancer Res. 2018;37(1):287. 30482232 10.1186/s13046-018-0934-9 PMC6258492

[54] Le F, Yang L, Han Y, Zhong Y, Zhan F, Feng Y, et al. TPL Inhibits the invasion and migration of drug-resistant ovarian cancer by targeting the PI3K/AKT/NF-kappaB-signaling pathway to inhibit the polarization of M2 TAMs. Front Oncol. 2021;11: 704001. 34381726 10.3389/fonc.2021.704001 PMC8350572

[55] Colozza G, Koo BK. Wnt/beta-catenin signaling: structure, assembly and endocytosis of the signalosome. Dev Growth Differ. 2021;63(3):199–218. 33619734 10.1111/dgd.12718 PMC8251975

[56] Chatterjee A, Paul S, Bisht B, Bhattacharya S, Sivasubramaniam S, Paul MK. Advances in targeting the WNT/beta-catenin signaling pathway in cancer. Drug Discov Today. 2021. 10.1016/j.drudis.2021.07.007. 34252612 10.1016/j.drudis.2021.07.007

[57] Yu X, Zhang X, Zhang Y, Jiang G, Mao X, Jin F. Cytosolic TMEM88 promotes triple-negative breast cancer by interacting with Dvl. Oncotarget. 2015;6(28):25034–45. 26325443 10.18632/oncotarget.4379 PMC4694813

[58] Herrera-Quiterio GA, Encarnación-Guevara S. The transmembrane proteins (TMEM) and their role in cell proliferation, migration, invasion, and epithelial-mesenchymal transition in cancer. Front Oncol. 2023;13:1244740. 37936608 10.3389/fonc.2023.1244740 PMC10627164

[59] Wang N, Ma T, Yu B. Targeting epigenetic regulators to overcome drug resistance in cancers. Signal Transduct Target Ther. 2023;8(1):69. 36797239 10.1038/s41392-023-01341-7 PMC9935618

[60] Flamant L, Roegiers E, Pierre M, Hayez A, Sterpin C, De Backer O, et al. TMEM45A is essential for hypoxia-induced chemoresistance in breast and liver cancer cells. BMC Cancer. 2012;12:391. 22954140 10.1186/1471-2407-12-391 PMC3519606

