# Supplementary material for: TMEM205 induces TAM/M2 polarization to promote cisplatin resistance in gastric cancer
Source: Gastric Cancer. 2024 Jun 8;27(5):998–1015. doi: 10.1007/s10120-024-01517-2 (PMC11335886; doi:10.1007/s10120-024-01517-2)
Supplement: Supplementary file 1 — Supplementary file1 (DOCX 795 KB) [file 10120_2024_1517_MOESM1_ESM.docx]

**Supplementary Figure：**

**
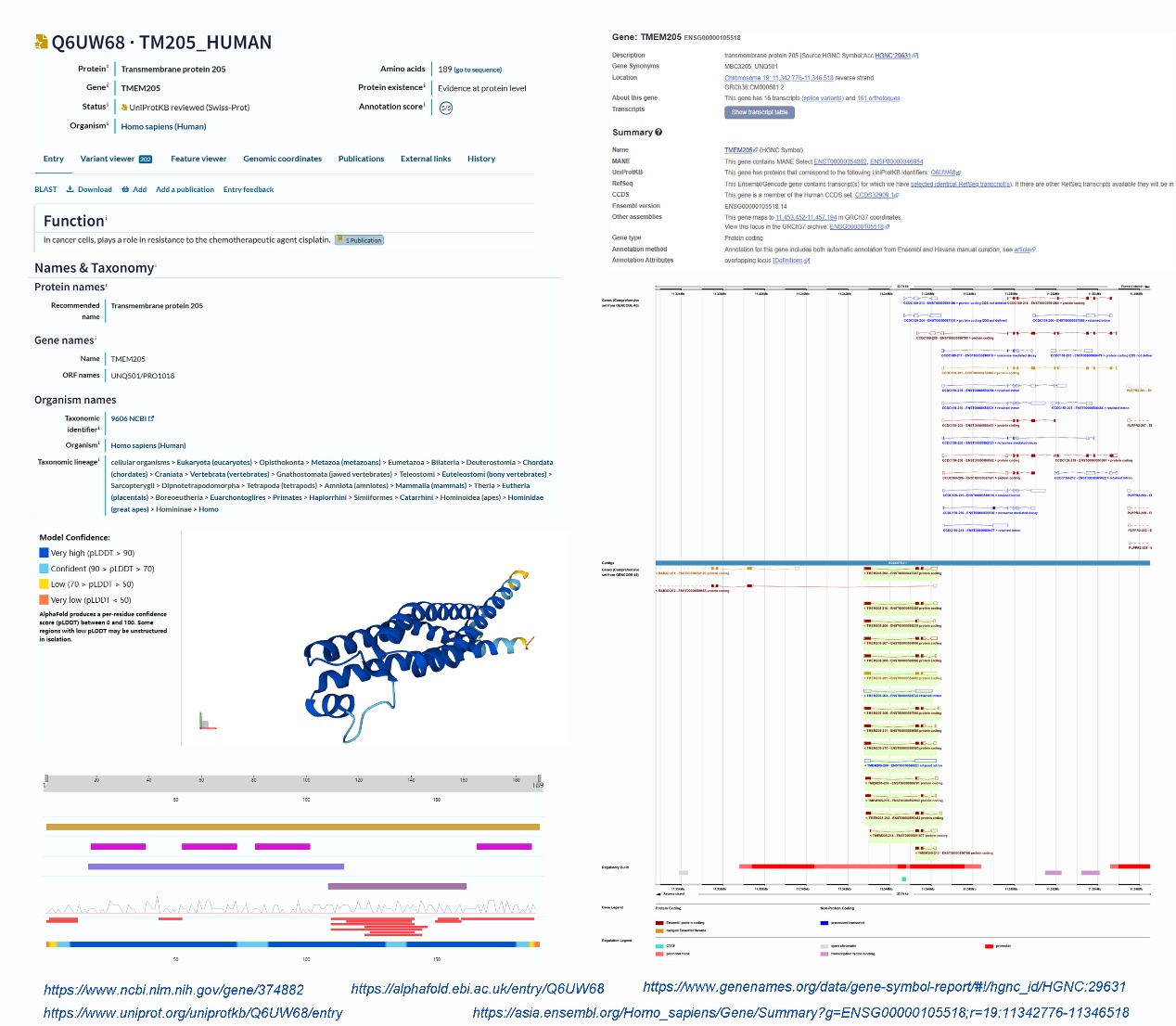
**

**Supplementary Figure 1**: **The molecular structure of TMEM205.**


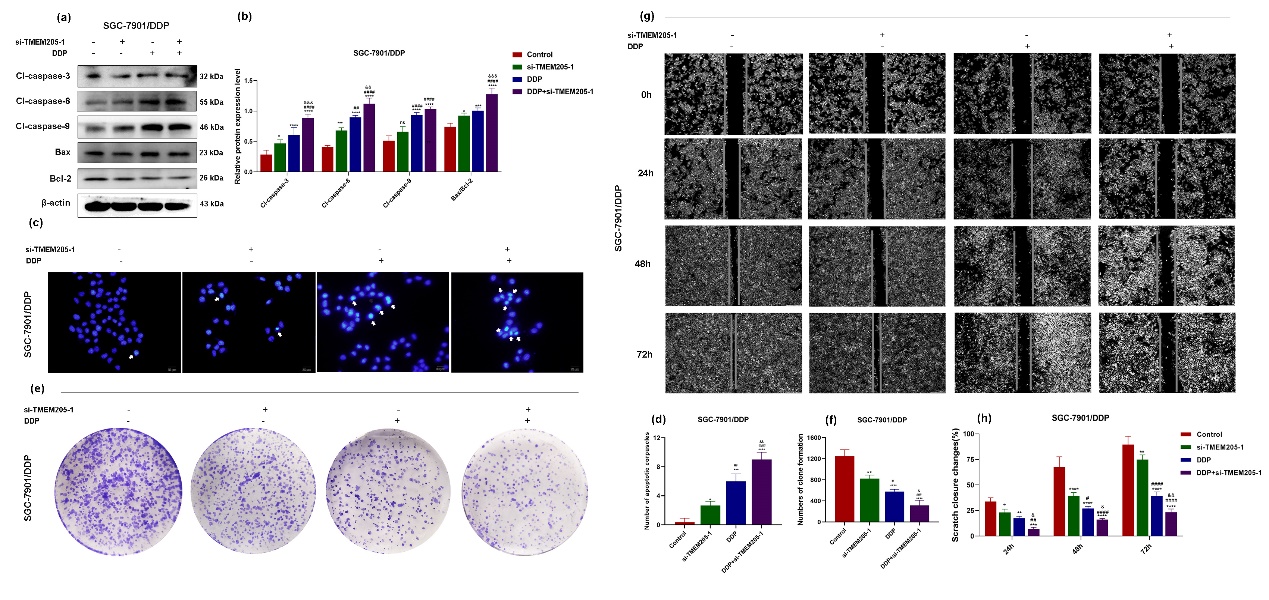


**Supplementary Figure 2**: **Combination of si-TMEM205-1 and DDP Promotes Apoptosis, Inhibits Proliferation, and Suppresses Migration Ability of SGC-7901/DDP Cells.** (WB and Hoechst 33342 staining showed that compared to the control group, both si-TMEM205-1 and DDP groups could induce apoptosis in the gastric cancer resistant cell line SGC-7901/DDP, with a more significant apoptotic effect observed when combined (a-d). Cloning and scratch assay results demonstrated that compared to the control group, both si-TMEM205-1 and DDP groups could inhibit the proliferative and migratory abilities of SGC-7901/DDP cells, with a more pronounced inhibitory effect observed when used in combination (e-h).)


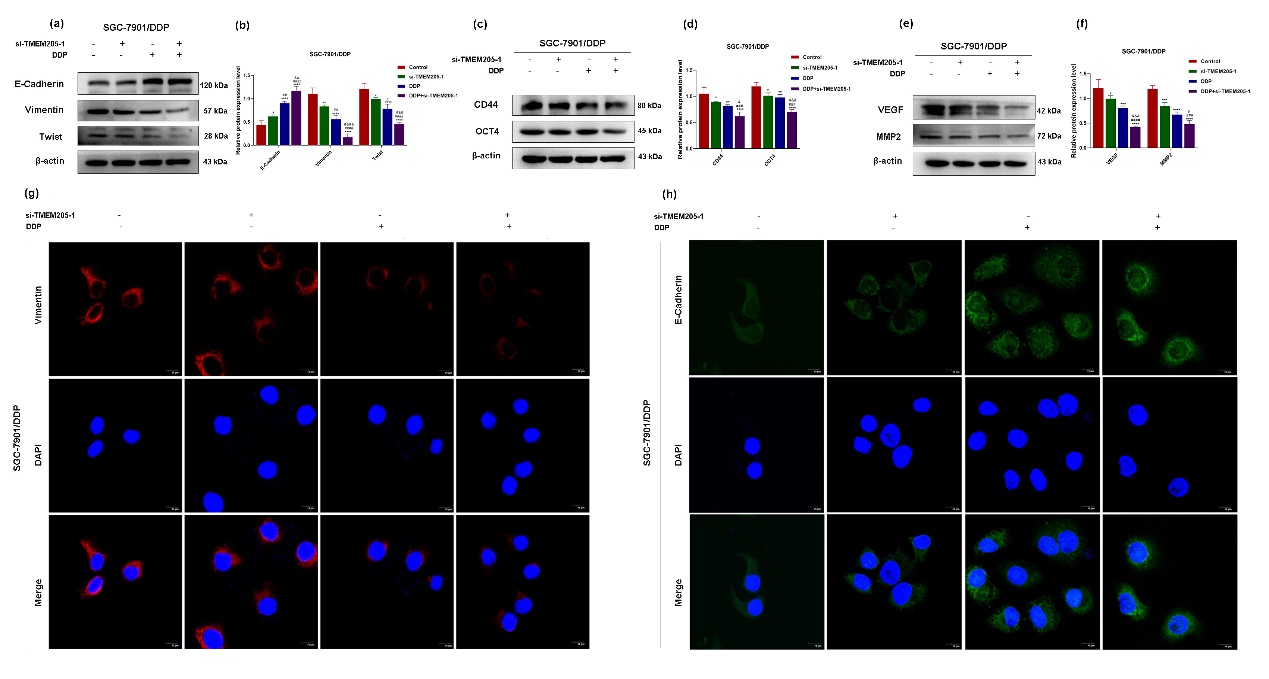


**Supplementary Figure 3:** **Combination of si-TMEM205-1 and DDP Suppresses EMT Process, Stemness, and Migration Ability of SGC-7901/DDP Cells.** (Western Blot results show that compared to the control group, both si-TMEM205-1 and DDP groups upregulate the expression of epithelial marker E-Cadherin and downregulate the expression of mesenchymal markers Vimentin and Twist in SGC-7901/DDP cells, with a more significant effect observed in the combination treatment group (a-b); immunofluorescence experiments corroborate these results (g-h). Western blot experiments also demonstrate that compared to the control group, both si-TMEM205-1 and DDP groups downregulate the expression of stem cell markers CD44 and Oct4, as well as angiogenesis-related proteins VEGF and MMP2 in SGC-7901/DDP cells, with a more pronounced inhibitory effect observed after combination treatment(c-f)).
